# Impact of Transgenic Wheat with *wheat yellow mosaic virus* Resistance on Microbial Community Diversity and Enzyme Activity in Rhizosphere Soil

**DOI:** 10.1371/journal.pone.0098394

**Published:** 2014-06-04

**Authors:** Jirong Wu, Mingzheng Yu, Jianhong Xu, Juan Du, Fang Ji, Fei Dong, Xinhai Li, Jianrong Shi

**Affiliations:** 1 Institute of Food Safety and Detection, Jiangsu Academy of Agricultural Sciences, Nanjing, China; 2 Key Lab of Food Quality and Safety of Jiangsu Province—State Key Laboratory Breeding Base, Nanjing, China; 3 Jiangsu Center for GMO evaluation and detection, Nanjing, China; 4 Institute of Crop Sciences, Chinese Academy of Agricultural Sciences, Beijing, China; University of Aveiro, Portugal

## Abstract

The transgenic wheat line N12-1 containing the *WYMV-Nib8* gene was obtained previously through particle bombardment, and it can effectively control the wheat yellow mosaic virus (WYMV) disease transmitted by *Polymyxa graminis* at turngreen stage. Due to insertion of an exogenous gene, the transcriptome of wheat may be altered and affect root exudates. Thus, it is important to investigate the potential environmental risk of transgenic wheat before commercial release because of potential undesirable ecological side effects. Our 2-year study at two different experimental locations was performed to analyze the impact of transgenic wheat N12-1 on bacterial and fungal community diversity in rhizosphere soil using polymerase chain reaction-denaturing gel gradient electrophoresis (PCR-DGGE) at four growth stages (seeding stage, turngreen stage, grain-filling stage, and maturing stage). We also explored the activities of urease, sucrase and dehydrogenase in rhizosphere soil. The results showed that there was little difference in bacterial and fungal community diversity in rhizosphere soil between N12-1 and its recipient Y158 by comparing Shannon's, Simpson's diversity index and evenness (except at one or two growth stages). Regarding enzyme activity, only one significant difference was found during the maturing stage at Xinxiang in 2011 for dehydrogenase. Significant growth stage variation was observed during 2 years at two experimental locations for both soil microbial community diversity and enzyme activity. Analysis of bands from the gel for fungal community diversity showed that the majority of fungi were uncultured. The results of this study suggested that virus-resistant transgenic wheat had no adverse impact on microbial community diversity and enzyme activity in rhizosphere soil during 2 continuous years at two different experimental locations. This study provides a theoretical basis for environmental impact monitoring of transgenic wheat when the introduced gene is derived from a virus.

## Introduction

Since the first successful genetically engineered (GE) plant was reported in 1983 [1], the planting area of transgenic crops has increased rapidly [2]. The global area cultivated commercially with transgenic crops has increased from 1.7 million ha in 1996 to 170.3 million ha in 2012 [3]. With the continued release and use of transgenic crops, there is a growing concern about their impact on the biota and soil microbial processes, such as nutrient cycling, and the potential risk of gene transfer from transgenic crops to indigenous soil microbes [4]–[5]. The microbes in rhizosphere soil play an important role in plant growth and development [6]–[7]. Transgenic crops planted in soil will inevitably interact with microorganisms such as bacteria, fungi, and actinomycetes [8]–[10]. Thus, transgenic crops may affect soil microbial population structure and quantity [11]–[13]. Additionally, root exudates have marked effects on soil microbial diversity and spatial distribution [14]–[15]. At this time, most studies of environmental risk assessment focused on transgenic Bt crops such as transgenic cotton, rice and maize containing the *Bt* gene [16]–[18]; these studies provided basic methods for environmental risk assessment for other crops.

Enzymes in the rhizosphere soil derived from animal, plant roots and soil microbial cell secretion and decomposition of residues are an important component of the soil ecosystem [19]. They play an important role in soil biochemical processes and directly affect soil fertility [19]. Urease is associated with nitrogen transformation in the soil, while sucrase is associated with soil organic matter, nitrogen and phosphorus contents, and dehydrogenase is associated with the redox ability of the soil [19]. Previous studies showed that transgenic plants might affect enzyme activities in rhizosphere soil [11], [20]–[21]. Therefore, it is important to investigate the impact of transgenic crops on rhizosphere soil enzyme activity when performing environmental safety risk assessments.

The first report of transgenic plants with virus resistance, expressing the coat protein of the *tobacco mosaic virus* (TMV) and delaying the development of disease, appeared in 1986 [22]. The same strategy was subsequently used to create resistance to a range of other viruses [23]–[24]. The exogenous genes of the transgenic virus-resistant crops are generally derived from the virus itself, including genes encoding coat protein and replicase [22]–[24]. Sequences derived from the genomes of plant viruses have been used to generate viral resistance in transgenic crop plants, but potential safety issues have been raised due to the environmental risks of transgenic plants with virus resistance, including heteroencapsidation, virus recombination, gene flow, synergism and effects on non-target organisms [25], [26].

Wheat yellow mosaic disease, caused by the wheat yellow mosaic virus (WYMV) at turngreen stage, is a serious illness affecting wheat in the middle and lower reaches of the Yangtze River region in China [27]–[28]. Disease-resistant variety breeding is one of the most cost-effective ways to control this disease through conventional wheat breeding. In recent years, conventional wheat breeding in combination with genetic engineering techniques has been applied to address wheat yellow mosaic disease, and some disease-resistant wheat lines have been cultivated. Using the particle bombardment method, genes from WYMV encoding replicase WYMV-Nib8 were transferred to the disease-sensitive variety Yangmai158 (Y158), and the disease-resistant transgenic wheat line named N12-1 was obtained by successive backcross with Y158 [29]. N12-1 showed stable and effective resistance to wheat yellow mosaic disease in a previous study [30].

Considering the above risks, transgenic virus-resistant wheat may affect the microbial community diversity in rhizosphere soil and change the population structure. Exogenous insertion of genes may also cause changes in the metabolic pathways of genetically modified crops and alter the composition of root exudates, resulting in changes in soil enzyme activity [31]. Thus, further studies on the impact on soil microbial community diversity and enzyme activities should be performed. In this study, environmental risk assessment of N12-1 was performed during 2 consecutive years of wheat cultivation under field conditions at two different experimental stations. The research involved primarily: (i) differences in soil microbial (bacterial and fungal) diversity in rhizosphere soil between N12-1 and Y158 using polymerase chain reaction–denaturing gradient gel electrophoresis (PCR-DGGE) and (ii) the activity of enzymes (urease, sucrase and dehydrogenase) in rhizosphere soil. In this report, we provide a theoretical basis for environmental transgenic wheat monitoring.

## Materials and Methods

### Ethics statement

In our study, the research samples were rhizosphere soils in the presence of transgenic and non-transgenic wheat. This presented no ethical issue.

### Plant materials and field trial

Transgenic wheat line N12-1 and its recipient Yangmai158 (Y158) provided by the Chinese Academy of Agricultural Sciences (CAAS) were applied in this study. N12-1, which contains the *WYMV-Nib8* gene from wheat yellow mosaic virus, can effectively control the WYMV disease transmitted by *Polymyxa graminis* at turngreen stage. Y158 was one of the most popular varieties in the middle and lower reaches of the Yangtze River region in China. However, it is sensitive to WYMV disease and the yield decreased significantly due to effects of this severe disease.

This study was performed at Luhe experimental station for transgenic crop, Jiangsu Academy of Agricultural Sciences (Luhe) and Xinxiang experimental station for transgenic crop, Henan Academy of Agricultural Sciences (Xinxiang). The physical and chemical properties of the soil are provided in Table 1. pH value, water content, available nitrogen, phosphorus potassium and organic matter content were determined by potentiometry method, alkali solution diffusion method, sodium bicarbonate method, ammonium acetate extraction method, potassium dichromate method, respectively [32]. The experiment was conducted in two successive growth seasons of wheat (October 2010-June 2011 and October 2011-June 2012) in the same field in which transgenic crops had never been planted. Each variety (line) had four blocks, each of which was 10×6 m. The materials were planted in a row with a row length of 6 m and row spacing of 0.3 m. Distance between plants was 3 cm within a row. Completely random design was applied to arrange the experiment performed in the field, and the wheat was subjected to conventional field management, that was 375 kg/ha of compound fertilizer (N:P_2_O_5_:K_2_O = 1∶0.4∶1) as base fertilizer and 225 kg/ha of urea as topdressing at seedling stage.

**Table 1:** Main physical and chemical properties of the soil from two experiment locations before planting.

Experiment station	Physical and chemical properties					
	pH	water (%)	available nitrogen (mg/kg)	available phosphorus (mg/kg)	available potassium (mg/kg)	organic matter (%)
Luhe	5.8	20.55	110.16	90.81	857.99	1.44
Xinxiang	8.5	4.92	70.39	28.26	863.69	0.68

### Soil sampling

Rhizosphere soil samples were collected in both years at Luhe and Xinxiang at four growth stages [seeding stage (SS), turngreen stage (TS), grainfilling stage (GS), maturing stage (MS)]. Rhizosphere soil was defined as the soil still attached to the roots after the roots were shaken by hand. For each sampling site, five wheat plants were selected to collect rhizosphere soil and each block contains five sampling site. Rhizosphere soil from the five sampling sites per block was mixed as a composite rhizosphere soil sample. The soil samples were sieved using a 20-mesh sieve and then stored at 4°C until further use, usually within one month before DNA extraction.

### Soil DNA extraction

Total community DNA was extracted from 0.5 g of rhizosphere soil using an UltraClean Soil DNA Isolation Kit (MoBio Lab, USA). DNA extraction was performed according to the manufacturer's protocol.

### PCR amplification of 16S and18S rDNA fragments for DGGE analysis

The 16S rDNA fragments of bacteria were amplified by using the primer pair GC338f (5′-CGCCGCGCGCGGCGGGCGGGGCGGGGGCACGGGGGGACTCCTACGGGAGGCAGCAG-3′, the sequence underlined was the GC clamp) and 518r (5′- ATTACCGCGGCTGCTGG -3′) as described by Bakke et al. [33]. High fidelity polymerase of KOD-Plus-Neo (Toyobo, Japan) was applied to perform PCR amplification and avoid mutations in the PCR product. Briefly, the reaction mixture consisted of 1 µl of template DNA (1–5 ng), 5 µl 10×PCR Buffer, 5 µl of 2 mM dNTPs, 3 µl of 25 mM MgSO_4_, 0.5 µl of 10 µM forward primer, 0.5 µl of 10 µM reverse primer, and 1 U of DNA polymerase, after which ddH_2_O was added to a final volume of 50 µl. The thermal cycling program was performed with an initial denaturation at 94°C for 5 min, followed by 35 cycles at 95°C for 15 sec, 58°C for 15 sec, and 68°C for 30 sec before the final extension at 68°C for 10 min. Products were checked by electrophoresis in 1% (wt/vol) agarose gels followed by ethidium bromide staining.

The 18S rDNA fragments of fungi were amplified by using the primer pair (GC-Fungi: 5′-CGCCCGCCGCGCCCCGCGCCCGGCCCGCCGCCCCCGCCCCATTCCCCGTTACCCGTTG-3′; NS1: 5′- GTAGTCATATGCTTGTCTC -3′, the sequence underlined was the GC clamp) as described by Das et al. [34]. The protocol for PCR amplification was similar as above. All products were purified before electrophoresis using a Cycle Pure Kit (Omega, USA).

### PCR-DGGE

DGGE analysis for 16S rDNA and 18S rDNA products was performed with the DCode System (Bio-Rad, USA). Polyacrylamide gels were composed of a denaturing gradient of 50–65% (bacteria) and 30–38% (fungi) urea, 0.17% (vol/vol) TEMED, 0.047% (wt/vol) ammonium persulfate, 6% acrylamide-N,N_-methylenebisacrylamide (37.5∶1) and 1×TAE. PCR products (up to 50 µl) were applied to the gel. DGGE was performed at 50 V in 1×TAE at 60°C for 12 h (bacteria) and at 50 V in 1×TAE at 60°C for 20 h (fungi), respectively. A silver staining method was used for the detection of DNA in DGGE gels.

Migration and intensity of DGGE bands were analyzed using Quantity One according to the manual. The bands that shared identical migration positions were considered to be the same species. Shannon's diversity index (H) of bacterial and fungal DGGE profiles was calculated with the following formula [35]:
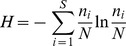
Simpson's diversity index (D) was calculated with the following formula:
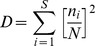
Evenness (E) was calculated with the following formula:
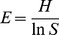

*n_i_* represented the square of individual peaks detected by Quantity One; *N* represented the square of all peaks in the same lane; *S* represented the number of bands in the same lane.

### Band sequencing

Visible bands in the fungi DGGE gel were picked with sterile tips and transferred into a 200 µl tube. Sterile ddH_2_O (50 µl) was added to the tube and the gel was pounded to pieces. The tubes with broken gels were incubated at room temperature overnight and then centrifuged at 12000 rpm for 5 min. The supernatant solution was used as the template for PCR, which was performed as described above or for fungi using primers without GC-clamps. The PCR products were purified (Omega, USA), ligated into pM19-T vector (Takara, Japan) and transformed into competent cells (*E coli* DH5α, Takara, Japan) according to the instructions of the manufactures and plated on LB solid medium with ampicillin. Positive clones were selected by PCR with primer pair of NS1 and Fungi (GC-Fungi without GC-clamps) and plasmids were extracted for sequencing (Invitrogen, Shanghai). All the sequences that have been sequenced successfully were submitted to GenBank (Accession numbers: KJ755390-KJ755404).

### Enzyme activity analysis

Activities of urease, sucrose and dehydrogenase were analyzed in this study. Urease and sucrose activities in soil were assayed using the method of Guan [36]: urease activity was determined by measuring the release of NH_3_ as mg.(g.d)^−1^, and sucrose activity was determined based on 3,5-dinitrosalicylic acid colorimetry as mg.(g.d)^−1^. Dehydrogenase activity was determined based on the reduction of triphenyltetrazolium chloride (TTC) to triphenylformazan (TPF), as described by Serra-Wittling et al. [37] with minor modifications, which was expressed as µg.(g.d)^−1^. The data were subjected to analysis of variance, and the means and standard deviations of four replicates were calculated.

### Statistical analysis

SPSS 16.0 was applied to determine whether the indices and enzyme activities differed between years, varieties and growth stages by ANOVA. PCA analyses were carried out based on band position and presence (presence/absence), and then the correlation matrix principal component analysis was performed by SPSS 16.0 [35]. Microsoft Excel 2003 was used to construct column diagrams.

## Results

### Impact of transgenic wheat on bacterial and fungal community diversity

**Figure 1:**
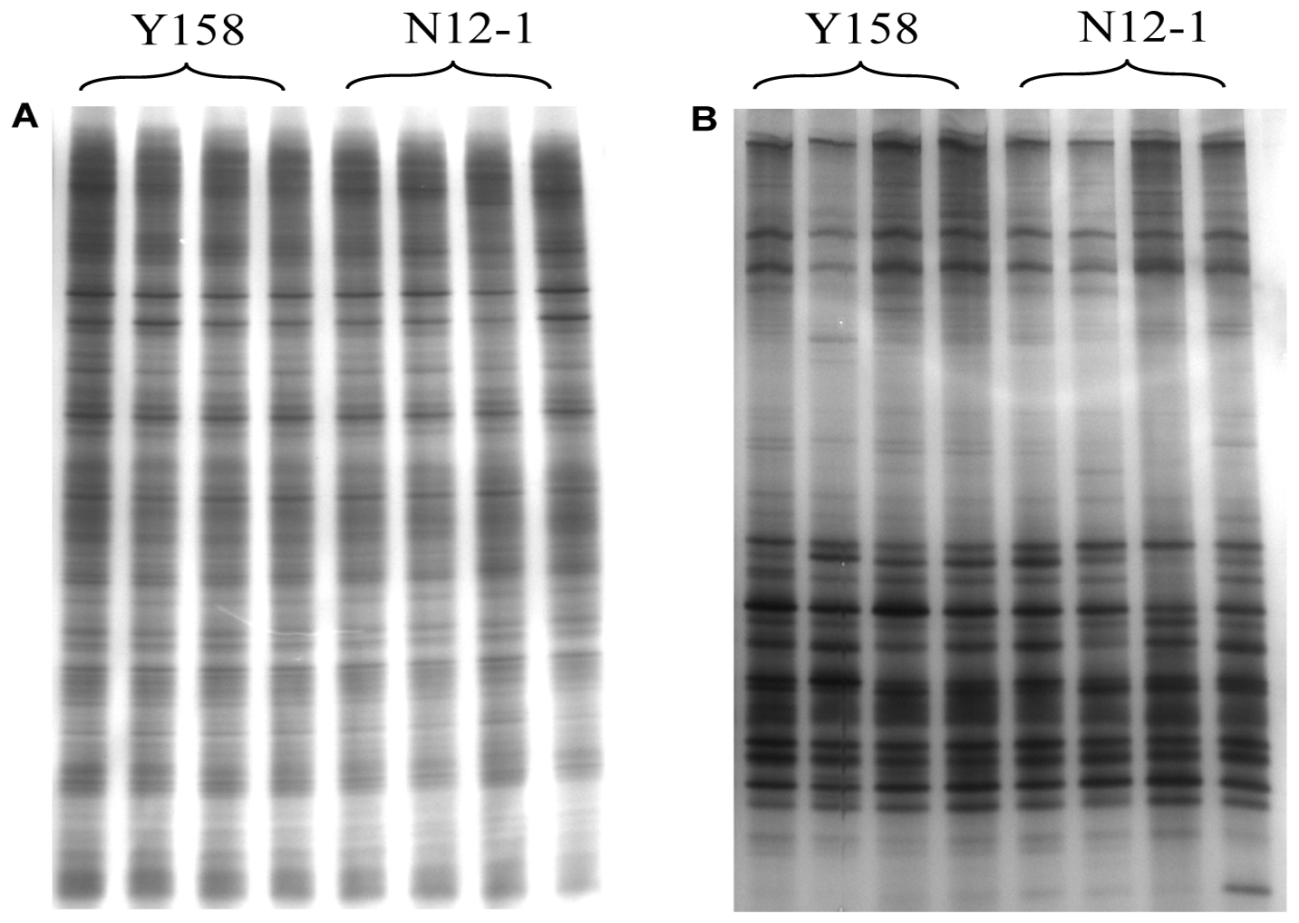
DGGE profiles of 16S rDNA and 18S rDNA fragments amplified from DNA extracted from rhizosphere soil of N12-1 and Y 158 at turngreen stage from Luhe experiment station in 2011. A: bacteria; B: fungus.

**Figure 2:**
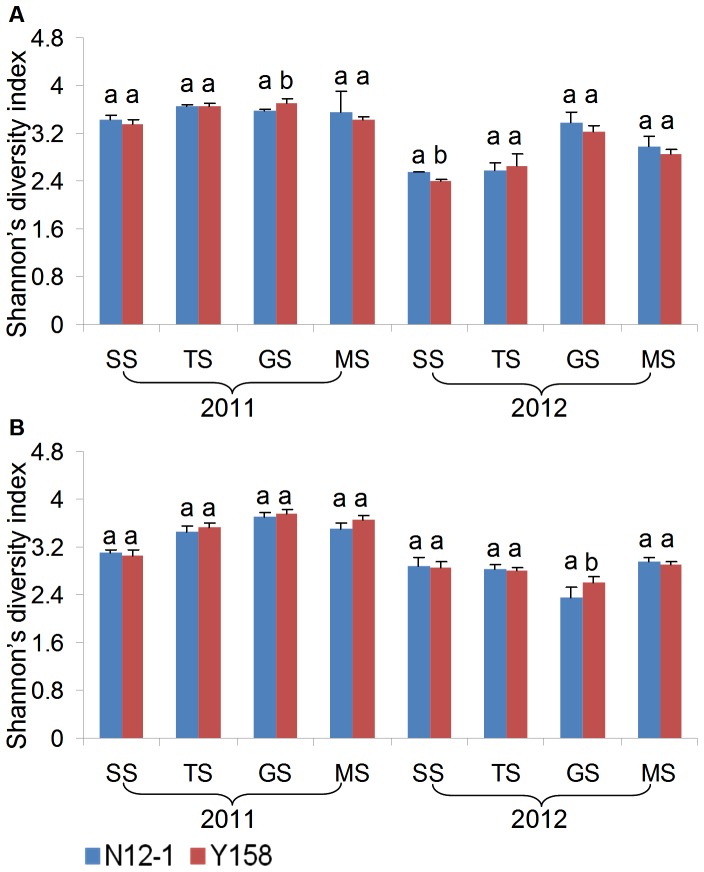
Shannon's index of bacterial communities at different growth stages. Error bars indicate standard errors (n = 4). Different letters above bars denote a statistically significant difference between the means of the fields. A: Luhe; B: Xinxiang. SS: seeding stage; TS: turngreen stage; GS: grainfilling stage; MS: maturing stage.

**Figure 3:**
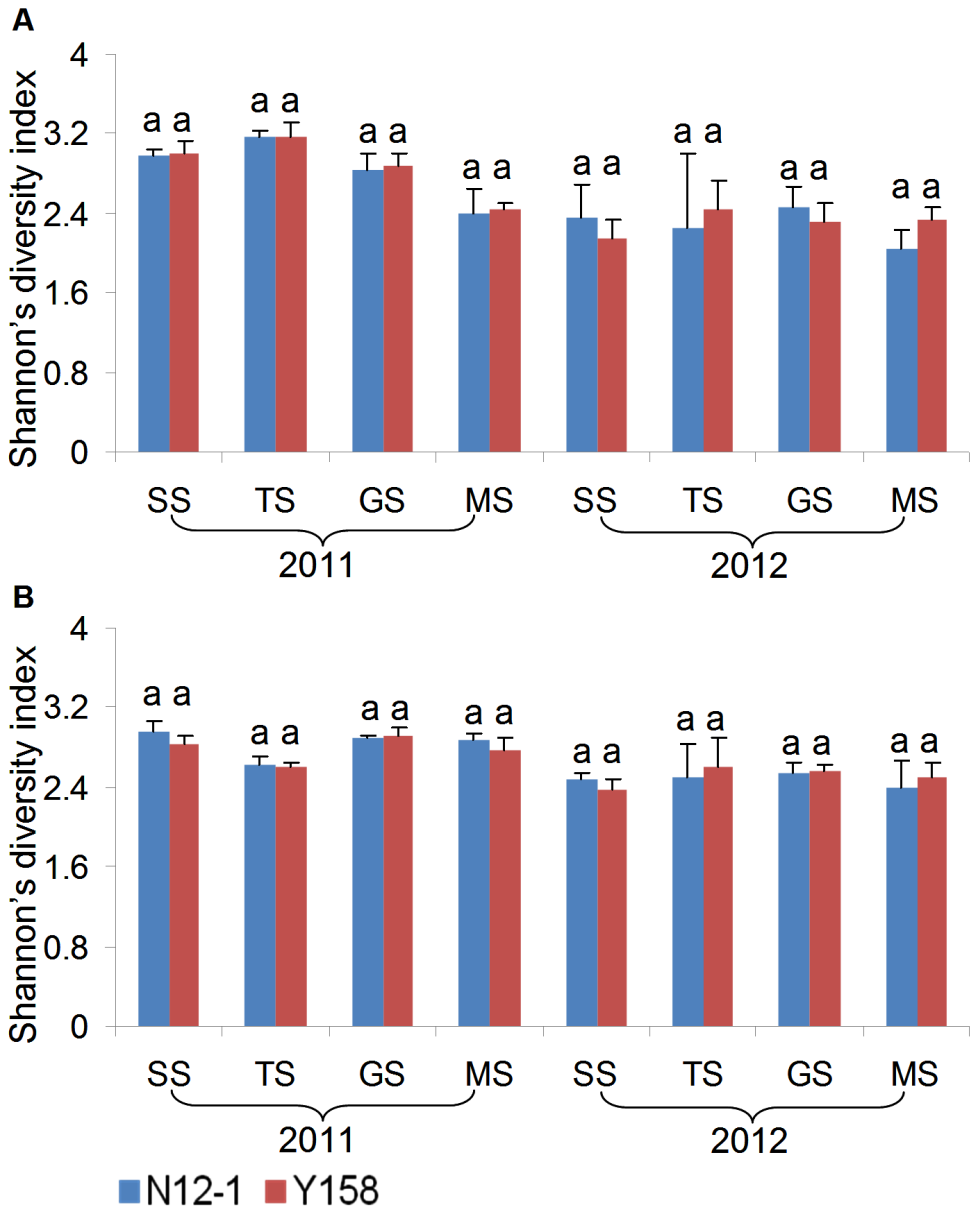
Shannon's index of fungi communities at different growth stages. Error bars indicate standard errors (n = 4). Different letters above bars denote a statistically significant difference between the means of the fields. A: Luhe; B: Xinxiang. SS: seeding stage; TS: turngreen stage; GS: grainfilling stage; MS: maturing stage.

One of the DGGE profiles of 16**S** rDNA and 18S rDNA fragments amplified from DNA extracted from rhizosphere soil was presented as figure 1. Three diversity indices (Shannon's, Simpson's, evenness) were used to analyze the bacterial and fungal DGGE profiles of the soil samples from Luhe and Xinxiang at four different growth stages in 2011 and 2012. For bacteria, the effect of wheat line on DGGE diversity indices was insignificant, except GS stage in 2011, SS in 2012 at Luhe and GS stage in 2012 at Xinxiang for Shannon's diversity index (Fig. 2). The Simpson's diversity index showed the same results as Shannon's diversity index (Table 2). For evenness, only one difference was found at the MS stage at Xinxiang in 2011 (Table 2). For fungi, the effect of wheat line on DGGE diversity indices was insignificant, except for SS in 2011 at Xinxiang and MS in 2012 at Luhe for Simpson's index (Fig. 3; Table 3).

**Table 2:** Simpson's index and Evenness of bacterial community.

Experiment station	Growth stage	Variety(line)	Simpson's index		Evenness	
			2011	2012	2011	2012
Luhe	SS	N12-1	0.04±0.00a	0.10±0.00a	0.95±0.01a	0.88±0.01a
		Y158	0.04±0.00a	0.12±0.00b	0.95±0.01a	0.86±0.02a
	TS	N12-1	0.03±0.00a	0.09±0.01a	0.84±0.01a	0.92±0.03a
		Y158	0.03±0.00a	0.08±0.02a	0.95±0.01a	0.93±0.01a
	GS	N12-1	0.03±0.00a	0.04±0.01a	0.95±0.00a	0.93±0.01a
		Y158	0.03±0.00b	0.05±0.01a	0.96±0.01a	0.93±0.02a
	MS	N12-1	0.04±0.00a	0.06±0.02a	0.99±0.10a	0.93±0.03a
		Y158	0.04±0.00a	0.07±0.00a	0.95±0.01a	0.90±0.02a
Xinxiang	SS	N12-1	0.05±0.01a	0.06±0.01a	0.93±0.03a	0.98±0.01a
		Y158	0.05±0.01a	0.06±0.01a	0.95±0.01a	1.00±0.01a
	TS	N12-1	0.04±0.01a	0.07±0.01a	0.93±0.02a	0.93±0.02a
		Y158	0.04±0.00a	0.07±0.01a	0.93±0.00a	0.94±0.02a
	GS	N12-1	0.03±0.00a	0.11±0.02a	0.95±0.01a	0.92±0.03a
		Y158	0.03±0.00a	0.08±0.01b	0.95±0.01a	0.96±0.01a
	MS	N12-1	0.04±0.01a	0.07±0.00a	0.93±0.02a	0.90±0.01a
		Y158	0.03±0.00a	0.07±0.01a	0.96±0.01b	0.89±0.02a

SS: seeding stage; TS: turngreen stage; GS: grainfilling stage; MS: maturing stage. The alphabets after the value represented the significance level of the index.

**Table 3:** Simpson's index and Evenness of fungi community.

Experiment station	Growth stage	variety(line)	Simpson's index		Evenness	
			2011	2012	2011	2012
Luhe	SS	N12-1	0.06±0.01a	0.11±0.03a	0.92±0.02a	0.94±0.02a
		Y158	0.06±0.01a	0.14±0.02a	0.94±0.01a	0.91±0.03a
	TS	N12-1	0.05±0.00a	0.04±0.06a	0.92±0.00a	0.78±0.23a
		Y158	0.05±0.01a	0.11±0.05a	0.91±0.01a	0.97±0.08a
	GS	N12-1	0.07±0.01a	0.10±0.02a	0.93±0.01a	0.95±0.02a
		Y158	0.07±0.01a	0.11±0.03a	0.93±0.01a	0.93±0.02a
	MS	N12-1	0.12±0.02a	0.16±0.03a	0.88±0.03a	0.86±0.04a
		Y158	0.11±0.01a	0.12±0.02b	0.89±0.04a	0.92±0.04a
Xinxiang	SS	N12-1	0.06±0.01a	0.10±0.01a	0.93±0.03a	0.87±0.02a
		Y158	0.07±0.00b	0.10±0.01a	0.93±0.01a	0.86±0.03a
	TS	N12-1	0.08±0.01a	0.09±0.03a	0.95±0.01a	0.97±0.02a
		Y158	0.08±0.00a	0.08±0.02a	0.95±0.01a	0.97±0.00a
	GS	N12-1	0.07±0.00a	0.08±0.01a	0.94±0.02a	0.96±0.01a
		Y158	0.07±0.01a	0.08±0.01a	0.93±0.01a	0.96±0.01a
	MS	N12-1	0.07±0.01a	0.12±0.04a	0.89±0.03a	0.88±0.04a
		Y158	0.07±0.01a	0.10±0.02a	0.91±0.02a	0.89±0.03a

SS: seeding stage; TS: turngreen stage; GS: grainfilling stage; MS: maturing stage. The alphabets after the value represented the significance level of the index.

### Principal component analysis of bacterial community diversity

**Figure 4:**
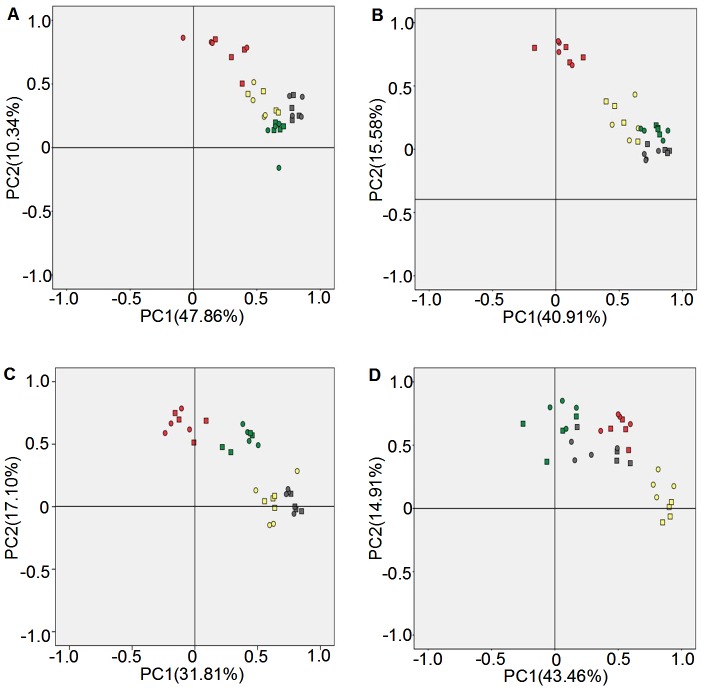
Principal component analysis of bacterial community diversities in rhizosphere soil. A: Luhe in 2011; B: Luhe in 2012; C: Xinxiang in 2011; D: Xinxiang in 2012. Square: N12-1; Round: Y158. Gray: seeding stage; Green: turngreen stage; Red: grainfilling stage; Yellow: maturing stage. Band position and presence (presence/absence) were used to carry out PCA analyses.

Principal components analysis (PCA) using both band position and presence/absence as parameters were performed to further analyze DGGE fingerprint profiles. For experiments conducted at Luhe, the contribution rates of the two principal components were 47.86% and 10.34% in 2011 (Fig. 4A) and 40.91% and 15.58% in 2012 (Fig. 4B), respectively. Different growth stages showed a distinct separation along the principal components axes, whereas different replications of experimental materials formed a cluster at the same growth stage. This was consistent with the result of Shannon's diversity analysis. In 2011, the first principal component axis clearly separated the GS and SS stage (Fig. 4A), but separated the GS and MS stage in 2012 (Fig. 4B). The second principal component axis clearly distinguished the GS stage in 2011 (Fig. 4A) and the GS stage in 2012 (Fig. 4B).

For experiments conducted at Xinxiang, the contribution rates of the two principal components were 31.81% and 17.10% in 2011 (Fig. 4C) and 43.46% and 14.91% in 2012 (Fig. 4D), respectively. Different growth stages also showed a distinct separation along the principal components axes, whereas different replications of experimental materials clustered together at the same growth stage. In 2011, the first principal component axis clearly separated the four growth stages (Fig. 4C), but separated the MS stage from the other three stages in 2012 (Fig. 4D). The second principal component axis clearly distinguished the TS and GS stages from the SS and MS stages in 2011 (Fig. 4C), and distinguished the MS stage in 2012 (Fig. 4D).

These PCA analysis results showed that growth stage played an important role in bacterial community diversity, rather than the presence of transgenic and non-transgenic wheat.

### Principal component analysis of fungal community diversity

**Figure 5:**
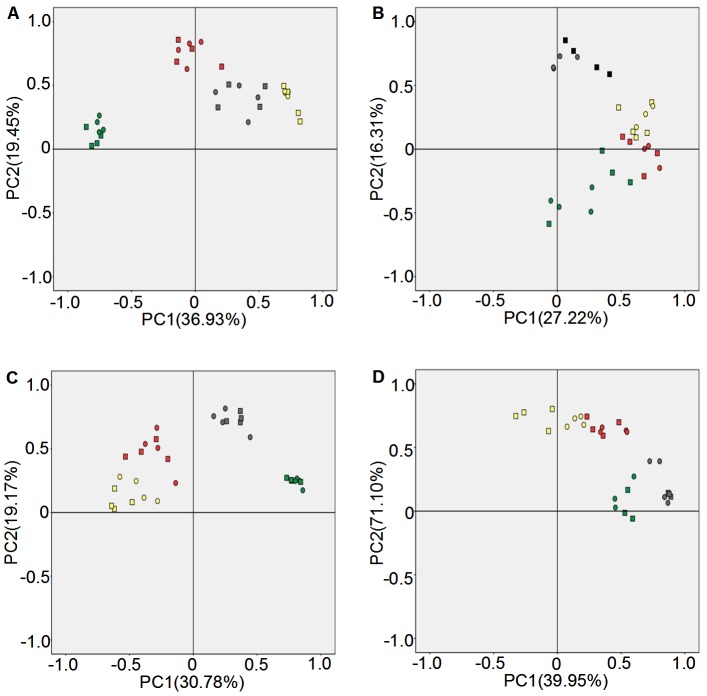
Principal component analysis of fungi communities diversity in rhizosphere soil. A: Luhe in 2011; B: Luhe in 2012; C: Xinxiang in 2011; D: Xinxiang in 2012. Square: N12-1; Round: Y158. Gray: seeding stage; Green: turngreen stage; Red: grainfilling stage; Yellow: maturing stage. Band position and presence (presence/absence) were used to carry out PCA analyses.

For experiments at Luhe, the contribution rates of the two principal components were 36.93% and 19.45% in 2011 (Fig. 5A) and 27.22% and 16.31% in 2012 (Fig. 5B), respectively. Different sampling times showed a distinct separation along the principal components axes, whereas different replications of experiment materials formed a cluster at the same sampling time. In 2011, the first principal component axis clearly separated the four growth stages (Fig. 5A), but separated SS and TS from GS and MS in 2012 (Fig. 5B). The second principal component axis clearly distinguished the TS stage in 2011 (Fig. 5A), and the SS and TS stage in 2012 (Fig. 5B).

For experiments at Xinxiang, the contribution rates of the two principal components were 30.78% and 19.17% in 2011 (Fig. 5C) and 39.95% and 17.10% in 2012 (Fig. 5D). Different sampling times also showed a distinct separation along the principal components axes, whereas different replications of experimental materials formed a cluster at the same sampling time. In 2011, the first principal component axis clearly separated the SS and TS stages (Fig. 5C), but separated the SS and MS stages in 2012 (Fig. 5D). The second principal component axis could not clearly distinguish any growth stage in 2011 (Fig. 5C), but could distinguish the MS and GS stages from the SS and TS stages in 2012 (Fig. 5D).

These PCA analysis results showed that fungal communities exhibited marked diversity at different growth stages, rather than between the transgenic line and non-transgenic wheat recipient.

### Band sequencing

**Figure 6:**
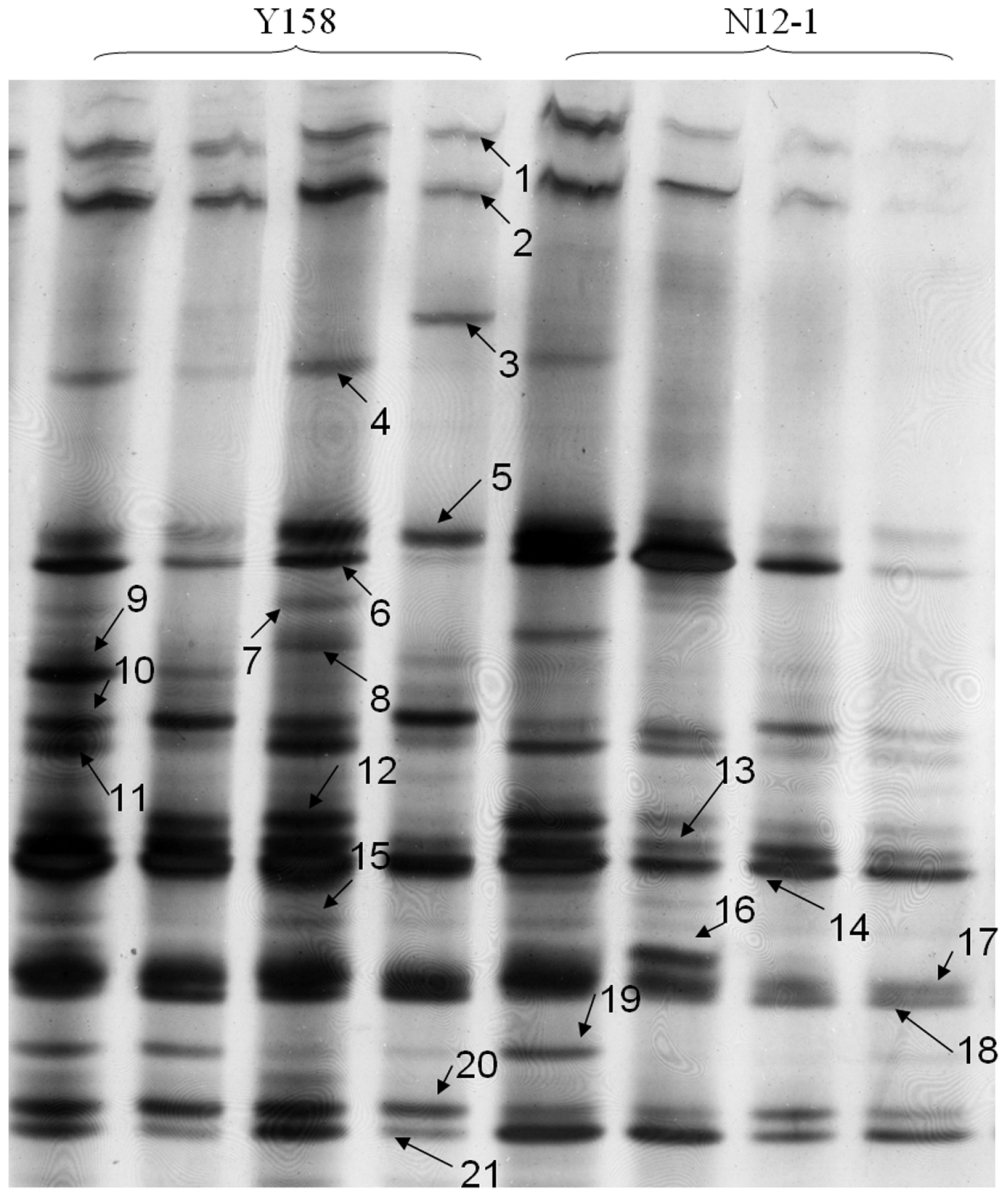
PCR-DGGE gel profile of fungi communities used for band sequencing. The numbers means different bands picked for sequencing.

A total of 21 visible bands from the DGGE gel of fungi from Luhe in 2011 were subjected to sequencing (Fig. 6), and 15 were sequenced successfully. Using NCBI BLAST, we found that most of the sequenced bands represented uncultured fungi. Others were partial 18S rRNA sequences of *Septoria dysentericae*, *Peziza varia*, *Saccobolus dilutellus*, *Polyozellus*, *Cochliobolus*, and *Myrothecium leucotrichym* (Table 4).

**Table 4:** Blast results of the bands from the DGGE gels of fungl community analysis.

No. of bands	Accession No.	Blast result	identity
1	GU214699.1	*Septoria dysentericae* strain CPC 12328 18S ribosomal RNA gene	100%
2	GQ330624.1	Uncultured *Mucorales* clone PR3 4E 28 18S ribosomal RNA gene	95%
3		Cannot be amplified	
4	AJ515922.1	Uncultured soil ascomycete partial 18S rDNA gene	100%
5	EU120944.1	Uncultured *Cystofilobasidiales* (aff. Guehomyces) clone Y9 18S ribosomal RNA gene	100%
6	AJ515941.1	Uncultured soil ascomycete partial 18S rDNA gene	99%
7		Cannot be amplified	
8	AY789390.1	*Peziza varia* strain ZW-Geo94-Clark 18S small subunit ribosomal RNA gene	99%
9	FJ176814.1	*Saccobolus dilutellus* isolate AFTOL-ID 1299 18S small subunit ribosomal RNA gene	97%
10	FO181499.1	Balen uncultured eukaryote partial 18S ribosomal RNA	80%
11	AY771600.1	Polyozellus multiplex isolate AFTOL-ID 677 18S small subunit ribosomal RNA gene	99%
12	GU190186.1	*Cochliobolus* sp. Enrichment culture clone NJ-F5 18S small subunit ribosomal RNA gene	100%
13		Cannot be amplified	
14	AJ515948.1	Uncultured soil ascomycete partial 18S rDNA gene	99%
15		Cannot be amplified	
16	AJ301992.1	*Myrothecium leucotrichym* 18S RNA gene	99%
17	JX159444.1	Uncultured *Filobasidium* clone Cegs 957 18S ribosomal RNA gene	99%
18	KC171701.1	Uncultured fungus isolate DGGE gel band f10 18S ribosomal RNA gene	100%
19		Cannot be amplified	
20		Cannot be amplified	
21	EU120947.1	Uncultured *Ascobolus* clone Y12 18S ribosomal RNA gene	99%

### Enzyme activity analysis

Urease, sucrase, and dehydrogenase activities in rhizosphere soil were applied as indicators for environmental risk assessment of transgenic wheat N12-1 in this study.

**Figure 7:**
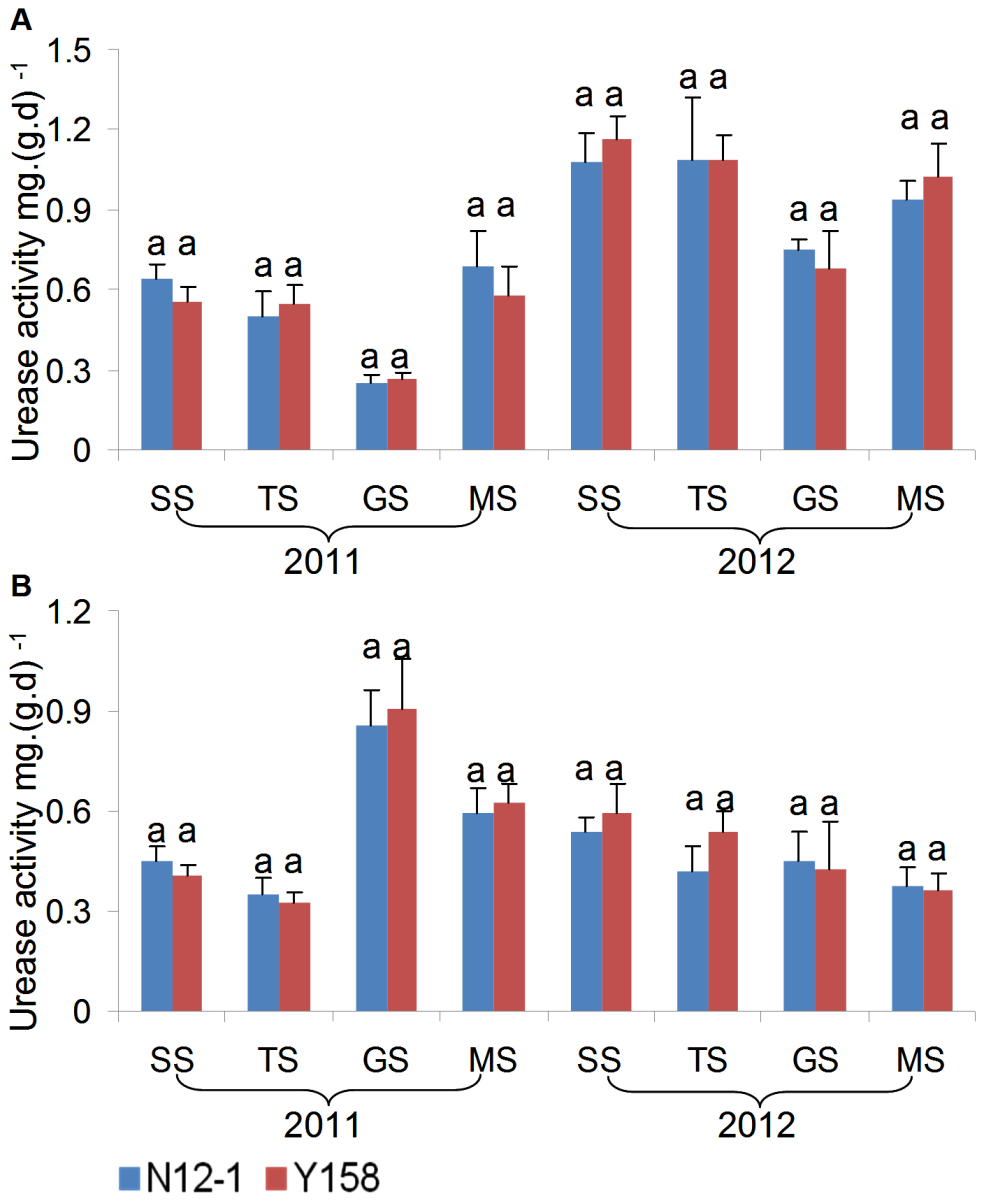
Urease activity in rhizosphere soil at different growth stages. Error bars indicate standard errors (n = 4). Different letters above bars denote a statistically significant difference between the means of the fields. A: Luhe; B: Xinxiang. SS: seeding stage; TS: turngreen stage; GS: grainfilling stage; MS: maturing stage.

**Figure 8:**
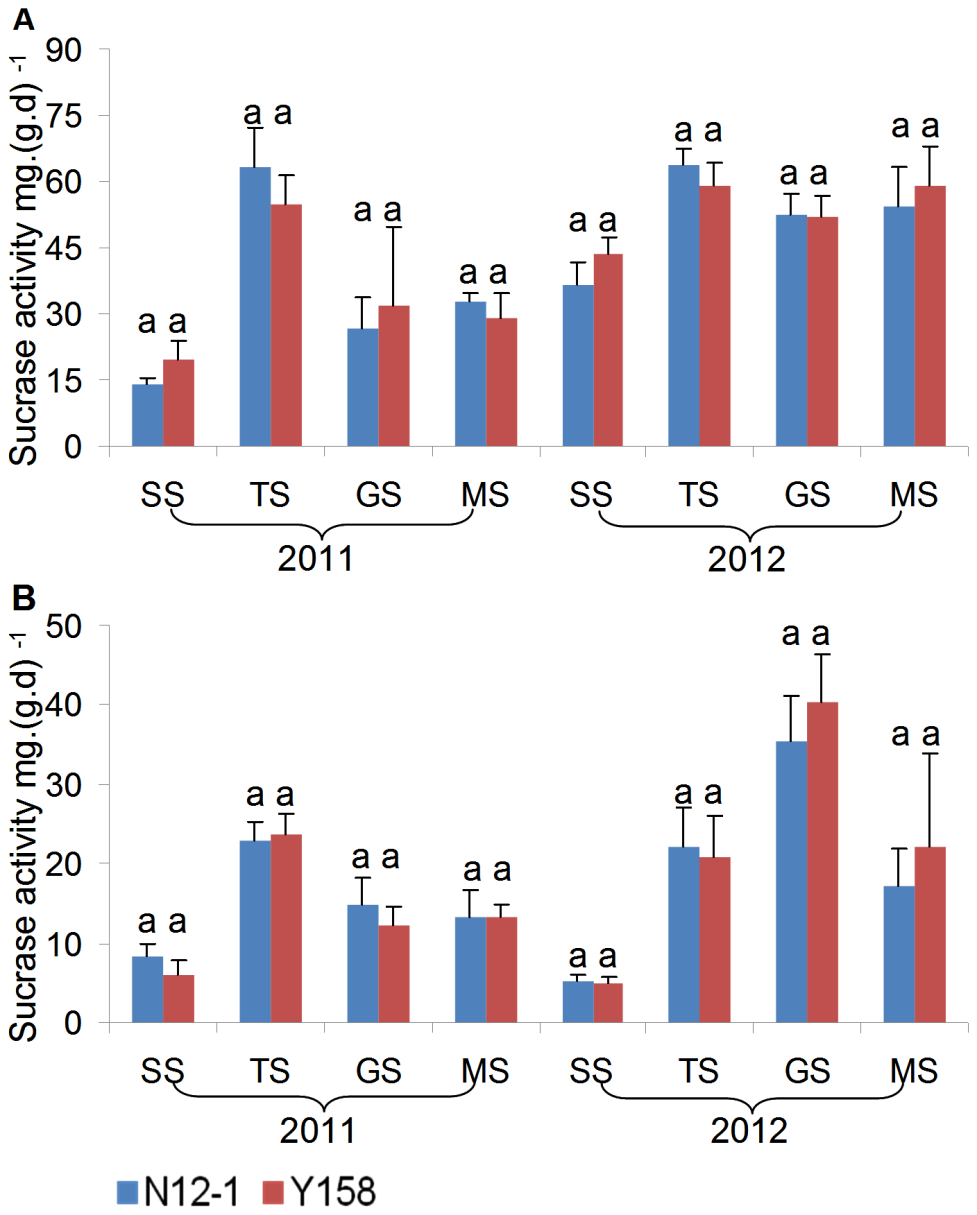
Sucrase activity in rhizosphere soil at different growth stages. Error bars indicate standard errors (n = 4). Different letters above bars denote a statistically significant difference between the means of the fields. A: Luhe; B: Xinxiang. SS: seeding stage; TS: turngreen stage; GS: grainfilling stage; MS: maturing stage.

**Figure 9:**
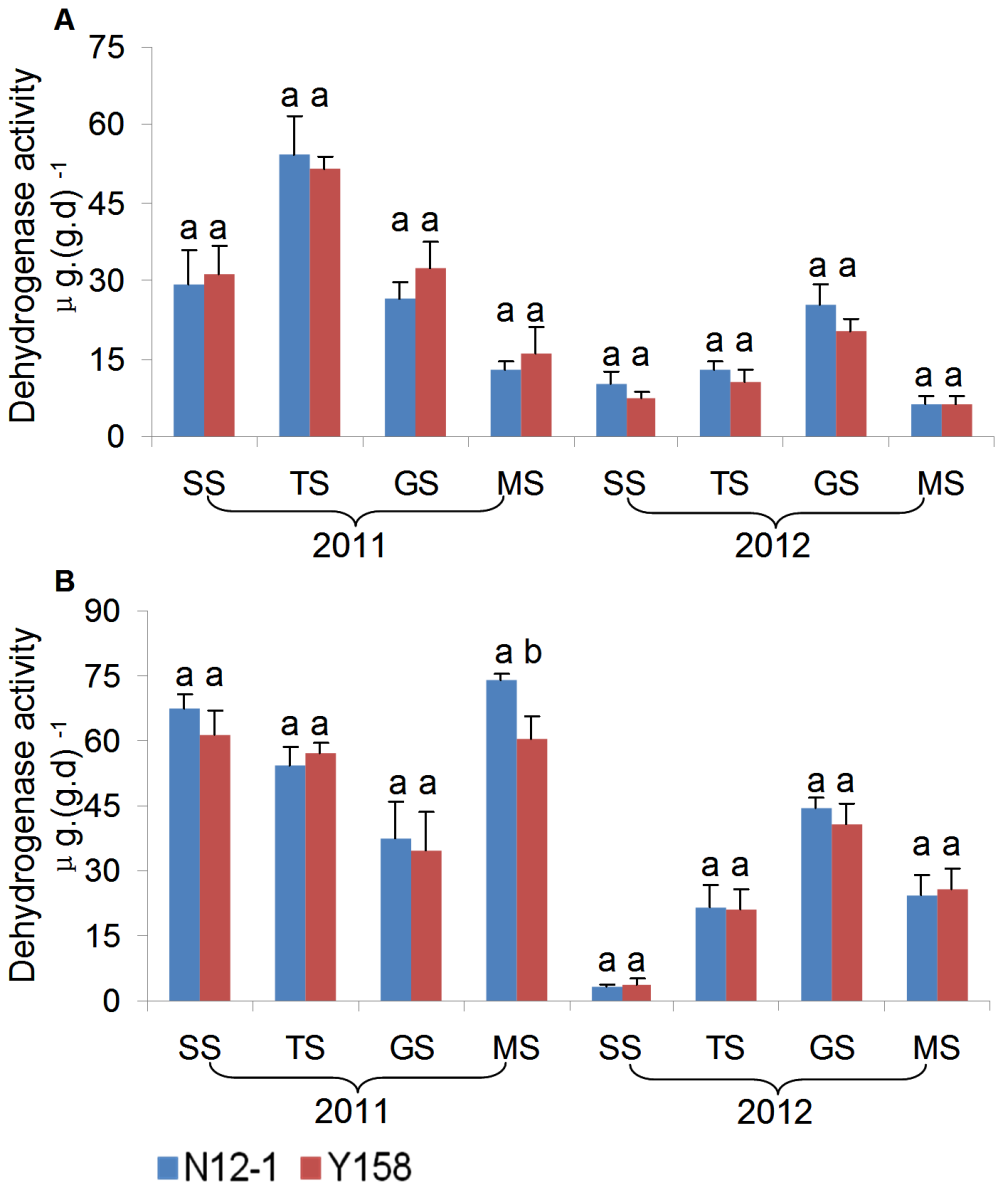
Dehydrogenase activity in rhizosphere soil at different growth stages. Error bars indicate standard errors (n = 4). Different letters above bars denote a statistically significant difference between the means of the fields. A: Luhe; B: Xinxiang. SS: seeding stage; TS: turngreen stage; GS: grainfilling stage; MS: maturing stage.

In general, there was no consistent significant difference in the enzyme activity between soils of transgenic wheat N12-1 and its recipient Y158 within the same growth stage during the 2 years. Only one significant difference in activity was observed; for dehydrogenase at the MS stage at Xinxiang in 2011. In 2011, the dehydrogenase activity in soil of N12-1 was significantly (*p*<0.05) higher than in soil of its recipient Y158 (Figs. 7–9). Significant differences were observed between years (*p*<0.01) and among growth stages (*p*<0.001) at both Luhe and Xinxiang, with the exception of dehydrogenase among growth stages at Xinxiang (*p*<0.25) (Table 5). These results showed that N12-1 had a minor impact on soil enzyme activities.

**Table 5:** Generalized Linear Mixed Model results for overall effects on enzyme activity.

Location	Enzyme	Effect	F Value	*p* Value
Luhe	Urease	Years	282.21	0.00
		Growth stage	36.89	0.00
		Variety (Line)	0.01	0.91
	Sucrase	Years	74.66	0.00
		Growth stage	33.54	0.00
		Variety (Line)	0.05	0.83
	Dehydrogenase	Years	82.20	0.00
		Growth stage	16.23	0.00
		Variety (Line)	0.03	0.87
Xinxiang	Urease	Years	6.38	0.01
		Growth stage	7.97	0.00
		Variety (Line)	0.103	0.75
	Sucrase	Years	11.45	0.00
		Growth stage	23.67	0.00
		Variety (Line)	0.10	0.75
	Dehydrogenase	Years	73.88	0.00
		Growth stage	1.40	0.25
		Variety (Line)	0.11	0.74

## Discussion

With the cultivation of more varieties of virus-resistant transgenic plants and large-scale planting, environmental impact monitoring after commercial release has attracted increasing attention from the scientific community and public [38], [39]. In soil, there are high microbial population densities and large numbers of microbial species that interact with the plants and surrounding environment and have an effect on the function of the soil ecosystem, such as the enzyme activity and physicochemical properties.

Soil microbial analysis has been used widely to evaluate the impact of various exogenous chemical or environmental pollutants (such as herbicides, fertilizers, heavy metals, et al.) on soil fertility and crop yields [7]. Therefore, monitoring changes in soil microbial populations will increase our understanding of the potential risks of introduction of exogenous genes to soil [4], [7]. In our study, two years and two locations of field research was performed to compare the impact of transgenic wheat with genes encoding replicase from WYMV on microbial population diversity in agricultural systems. One of the major outcomes was that transgenic insertion did not significantly alter bacterial or fungal population diversity at each growth stage; however, growth stage and planting year had important effects on microbial diversity. This result was similar to the findings of Meyer et al. [40]. In that study, the authors found that the effects of GM wheat on plant-beneficial root-colonizing microorganisms are minor and not of ecological importance. Lupwayi et al. reported that glyphosate-resistant wheat–canola rotations under low-disturbance direct seeding and conventional tillage did not affect the functional diversity of rhizosphere soil bacteria in 18 of 20 site-years [20]. The observation that certain growth stages (mainly SS and GS) showed differences between transgenic and non-transgenic wheat may be due to inconsistencies in the soil at seeding time, and at later growth stages the temperature and humidity increased rapidly. The differences between years and growth stages indicated that the diversity of bacteria and fungi might be affected by various environmental factors, such as temperature, humidity, and light. In studies of GM crops against virus, there was no significant difference between microbial communities with transgenic or non-transgenic watermelon resistant or cucumber green mottle mosaic virus (CGMMV), but significant changes in the microbial community were observed during the growing season [41]. Transgenic tomato resistant to cucumber mosaic virus (CMV) had no effect on the variation of soil microbial communities, in which soil position and environmental factors played more dominant roles [42]. Also, non-environmental factor, such as root exudates, may also play an important role for diversity changes of bacteria and fungi between years and growth stages [14]–[15]. Fang et al. thought that bacterial communities differed due to changes in root exudates quantity and composition by developing corn plant, which select different bacterial groups during root colonization [43]; Donegan et al. have speculated that the reason for the different in the communities of genetically modified plants is due to differences in the root exudates patterns of these plants [44]. However, Wei et al. reported the opposite result [45]. In a transgenic alfalfa study performed using the cultivation-dependent plating method, statistically significant differences in densities of rhizospheric bacteria between transgenic and non-transgenic alfalfa clones were observed for ammonifying bacteria, cellulolytic bacteria, rhizobial bacteria, denitrifying bacteria and *Azotobacter* spp. [46]. These results indicated that transgenic crops containing a viral gene conferring resistance to viral disease had little effect on soil microbial diversity (excluding a small number of studies) compared with non-transgenic crops. Transgenic wheat also had no adverse effects on soil biological indicators, such as *Folsomia candida* [47] and earthworm [48]. Duc et al. found that GM wheat with race-specific antifungal resistance against powdery mildew (Pm3b), and two with nonspecific antifungal resistance, had no impact on the soil fauna community (mites, springtails, annelids, and diptera). However, sampling date and location significantly influenced the soil fauna community and decomposition processes [49].

Soil enzymes in the soil nutrient cycle and energy transfer play an important role in soil ecology, and are derived mainly from soil microbial populations. Many studies have used soil enzymes as indicators of soil microbial activity and fertility [50]–[52]. In our study, urease, sucrase and dehydrogenase were used as indicators of the impact of transgenic wheat on soil quality. The results showed no significant difference in enzyme activity in rhizosphere soil between transgenic and non-transgenic wheat at each growth stage at two locations in 2 years, excluding dehydrogenase during the maturing stage at Xinxiang in 2011. In other studies of transgenic crops, there was no consistent significant difference in soil enzymes between transgenic and non-transgenic plants, but there were differences among seasons and crop varieties [53]–[54]. These results are consistent with our study. In other studies, some enzymes showed significant differences between transgenic and non-transgenic plants [11], [20]–[21]. There have been no previous studies of soil enzyme activities of transgenic wheat. Thus, our results should be confirmed in future studies and at more experimental locations. Additionally, other types of transgenic wheat, such as insect-resistant and stress-tolerant varieties, should be used to perform risk assessments.

Due to the complexity of DGGE profiles, several bands can be difficult to identify visually, and different bands represent different microbes. These issues make it difficult to compare varieties. Thus, the combination of DGGE and cloning sequencing methods is often used to investigate the impact of transgenic plants on microorganisms in rhizosphere soil [55], [56]. In our study, most of the bands from fungi DGGE gels represented uncultured fungal taxa. This is in agreement with the fact that only ∼1% of microbes in soil can be artificially cultured and identified [57].

With the development of sequencing technology, the way we study microbial communities has been changed. Traditionally, the study of genes from natural environments included cloning DNA into a vector, inserting that vector into a host, screening, and Sanger sequencing. Sequence-by-synthesis methods provide faster, cheaper, and simpler methods for (meta)genome sequence that bypass the PCR amplification bias, cloning bias and labor-intensive Sanger method [58]. Currently, massively parallel high-throughput pyrosequencing methods can process hundreds of thousands of sequences simultaneously [59]. Fierer et al. used metagenomic and small subunit rRNA analyses to study the genetic diversity of bacteria, archaea, fungi, and viruses in soil [60]; Uroz et al. used functional assays and metagenomic analyses to reveal difference between the microbial communities [61]. Li et al. analyzed the impact on bacterial community in midguts of the asian corn borer larvae by transgenic *Trichoderma* strain overexpressing a heterologous *chit42* gene with chitin-binding domain by using 16s rRNA library. All above studies have used the next generation sequencing technology [62]. Now, this technology is being adopted to study the microbial community in rhizosphere soil of transgenic plants gradually [62].

In conclusion, our study has produced weak evidence for the effect of virus-resistant transgenic wheat on soil microbial community diversity and enzyme activities. The community structure was markedly affected by natural variations in the environment related to wheat growth stage and planting year. Little difference was observed in bacterial and fungal communities in the presence of the wild-type Y158 or the transgenic line N12-1. This requires further investigation using extended field observations involving more varieties for more years. Based on this information, we can determine whether the altered composition is attributable to the presence of transgenic crops, or is simply part of the variation driven by the presence of different genotypes [63]. These studies should also involve more soil types and longer-term monitoring to account for the variability of the natural environment.
